# Mortality and associated factors of patients with extensive drug-resistant tuberculosis: an emerging public health crisis in China

**DOI:** 10.1186/s12879-018-3169-7

**Published:** 2018-06-07

**Authors:** Chengli Bei, Manjiao Fu, Yao Zhang, Hebin Xie, Ke Yin, Yanke Liu, Li Zhang, Bangruan Xie, Fang Li, Hua Huang, Yuhong Liu, Li Yang, Jing Zhou

**Affiliations:** 1 Changsha Central Hospital, Changsha, Hunan China; 2 Beijing Innovation Alliance of TB Diagnosis and Treatment, Beijing, China; 3 Wuhan Medical Treatment Center, Wuhan, Hubei China; 4 The Third People’s Hospital of Hengyang, Hengyang, Hunan China; 5 The Second People’s Hospital of Chenzhou, Chenzhou, Hunan China; 6 China Center on TB, China CDC, Beijing, China

**Keywords:** XDR-TB, Mortality, Survival analysis, Risk factors, New case, Transmission

## Abstract

**Background:**

Limited treatment options of extensive drug-resistant tuberculosis (XDR-TB) have led to its high mortality worldwide. Relevant data about mortality of XDR-TB patients in literature are limited and likely underestimate the real situation in China, since the majority of patients with XDR-TB are lost to follow-up after discharge from TB hospitals. In this study, we sought to investigate the mortality and associated risk factors of Human Immunodeficiency Virus (HIV)-negative patients with XDR-TB in China.

**Methods:**

All patients who were diagnosed with XDR-TB for the first time in four TB care centers across China between March 2013 and February 2015 were consecutively enrolled. Active tracking through contacting patients or family members by phone or home visit was conducted to obtain patients’ survival information by February 2017. Multivariable Cox regression models were used to evaluate factors associated with mortality.

**Results:**

Among 67 patients enrolled, the mean age was 48.7 (Standard Deviation [SD] = 16.7) years, and 51 (76%) were men. Fourteen patients (21%) were treatment naïve at diagnosis indicating primary transmission. 58 (86.8%) patients remained positive for sputum smear or culture when discharged. During a median follow-up period of 32 months, 20 deaths occurred, with an overall mortality of 128 per 1000 person-years. Among patients who were dead, the median survival was 5.4 months (interquartile range [IQR]: 2.2–17.8). Seventeen (85%) of them died at home, among whom the median interval from discharge to death was 8.4 months (IQR: 2.0–18.2). In Cox proportional hazards regression models, body mass index (BMI) < 18.5 kg/m^2^ (adjusted hazard ratio [aHR] *=* 4.5, 95% confidence interval [CI]: 1.3–15.7), smoking (aHR *=* 4.7, 95%CI:1.7–13.2), or a clinically significant comorbidity including heart, lung, liver, or renal disorders or auto-immune diseases (aHR *=* 3.5, 95%CI*:* 1.3–9.4), were factors independently associated with increased mortality.

**Conclusion:**

Our study suggested an alarming situation of XDR-TB patients in China with a sizable proportion of newly transmitted cases, a high mortality rate, and a long period in community. This observation calls for urgent actions to improve XDR-TB case management in China, including providing regimens with high chances of cure and palliative care, and enhanced infection control measures.

## Background

Tuberculosis (TB) is one of the top 10 causes of human death [1]. Drug-resistant (DR) TB poses a particular challenge, in which extensively drug-resistant tuberculosis (XDR-TB) is the most severe form. Based on 2017 Global Tuberculosis Report [1], more than 100 countries reported cases of XDR-TB in 2016, the rate of treatment success of XDR-TB were reported merely 30% in 2014. Although 89 territories and countries had started using bedaquiline and 54 had used delamanid by June 2017 to improve outcomes for Multi-drug resistant tuberculosis (MDR-TB) /XDR-TB, the mortality of XDR-TB reported from treatment cohorts were still above 30% [1–5]. The high treatment-associated costs are unsustainable in the middle-income and low-income countries that account for over 95% of tuberculosis related deaths [6]. Meanwhile the “transmitted resistance”-XDR-TB has also well described and confirmed throughout the world [7, 8].

China has the third largest TB burden in the world [1, 9]. In 2016, 895,000 TB cases were newly diagnosed, representing 16% of new cases worldwide. The prevalence of MDR-TB is 6.6% among Chinese patients with TB. In addition, based on a 2007 national survey, around 8% of MDR-TB cases were XDR-TB [10]. Survival situation of Chinese patients with XDR-TB is a crucial parameter to understand the related disease burden. However, relevant data in the literature are limited and likely underestimate the real situation. A multicenter survey in China found that XDR-TB mortality was very low (4.73%, 8/169) [11]; another study in Shanghai also found that the mortality rate of XDR-TB was in a low level (5.3%), and 7.4% was defaulted within 2~ 3 years’ treatment [12]. In addition to the lower incidence of Human Immunodeficiency Virus (HIV)-TB coinfections in China, We assume that the main reason for potential under-reported death for XDR-TB in China is the majority of patients with XDR-TB are lost to follow-up after discharge from TB hospitals. Since China had started using bedaquiline for MDR/XDR-TB patients until February 2018, many patients discharged from hospital while still sputum smear/culture positive because of limited therapeutic options and the high treatment-associated costs. For instance, Cui H et al. reported that 50% of patients with XDR-TB were not traceable after discharge from a TB referral hospital in Beijing [3]. Mortality of Chinese patients with XDR-TB has not been assessed using active tracking to address the issue of losing patients to follow-up.

With this study, we sought to evaluate the mortality of HIV-negative patients with XDR-TB and its related influencing factors through active tracking in four TB specialized hospitals in China.

## Methods

### Patients enrollment

All patients admitted into four TB specialist hospitals (Changsha Central Hospital, Wuhan Treatment Center, the Third People’s Hospital of Hengyang, and the Second People’s Hospital of Chenzhou) and initially diagnosed as XDR-TB by drug susceptibility testing (DST) between March 2013 and February 2015 were included into this study. Patients with previous confirmed XDR-TB and/or HIV-positive were excluded.

### Ethical approval

All patients’ information was routinely recorded and collected by attending physicians. This study was approved by the Ethical Review Committee of responsible units (Changsha Central Hospital) and we had received ethics committee approval for verbal consent. We obtained verbal informed consent from all subjects on the phone, if the patient died when we tracked, the verbal consent was received from the legal representative or next of kin of the deceased patients.

### Tracking and data collection

**Fig. 1:**
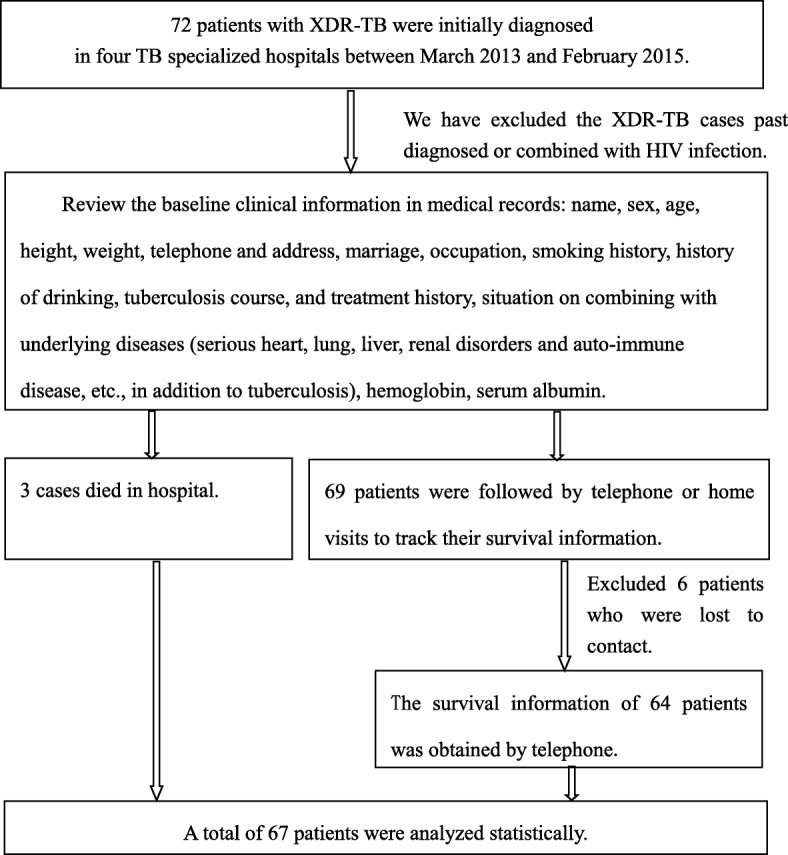
The enrollment and tracked process of study population of XDR-TB cases in four TB specialized hospitals, March 2013~February 2015

After the patients enrollment, Demographic and clinical information (including name, sex, age, height, weight, telephone and address, marriage, occupation, smoking history, history of drinking, tuberculosis course and treatment history, situation on combing with underlying diseases, hemoglobin, serum albumin) were obtained from medical record review. Effort was made to assess the vital status online for all patients with XDR-TB until February 2017. For the cases medical records not comprehensive, a phone interview or home visit was conducted. The end time of tracking of the patients who survived was February 16, 2017, and the death time of the dead patients. The end events of tracking was death of all deaths. The patient enrollment and tracked process is illustrated in Fig. 1.

### Definition

XDR-TB was defined by resistance to isoniazid, rifampicin, and at least one injectable agent (i.e., amikacin, kanamycin, capreomycin) and any of the fluoroquinolones [13, 14]. New TB cases were TB patients who had denied having any previous anti-TB treatment or history of more than 30 days of anti-TB treatment. Retreatment TB cases were defined as TB patients who had been accepting TB treatment for more than 30 days or who had recorded evidence of prior treatment in the surveillance database or case reports [15, 16].

### Statistical analysis

The baseline clinical features of XDR-TB patients were analyzed by descriptive analysis. Cox proportional hazards regression model was used to evaluate the association between baseline laboratory and clinical variables and death. The method used was “Backward:LR”; the probability stepwise was “entry = 0.05” and “removal = 0.1.” The Kaplan-Meier method was used to map the survival curves. SPSS software (version 22.0) was used to perform all statistical analyses, with a “*P* < 0.05” as the criterion for indicating statistical significance.

## Results

### Characteristics of enrolled patients with XDR-TB

Average age at admission was (48.7 ± 16.7) years (range, 19–99 years), and the average body mass index (BMI) was 18.9 ± 3.7 kg/m^2^ (range, 13.4–30.7 kg/m^2^), 51 (76%) patients were men, and 42 (63%) were off-site-referral patients. Of all patients, 79% (53/67) had a history of TB treatment, and the remaining 21% (14/67) were new TB cases.

### Outcomes of XDR-TB patients

Among the 67 patients, 3 patients died during hospitalization, 6 patients discharged from the hospital while sputum smear/culture converted to negative, and 58 (86.8%) patients remained positive for sputum smear or culture when discharged. A phone interview was conducted on the 64 patients who were still alive at discharge in February 2017.

**Fig. 2:**
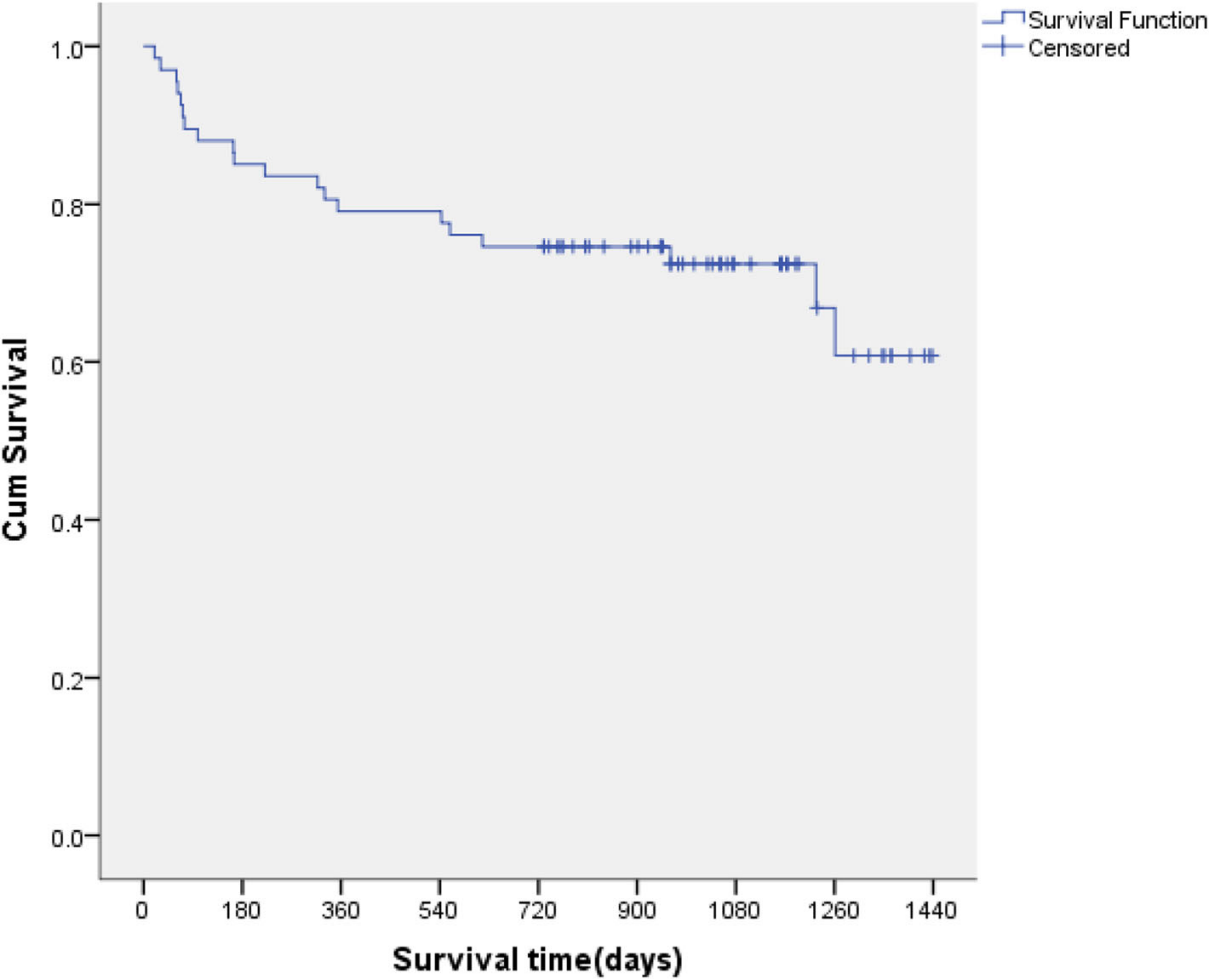
The survival curve of 67 XDR-TB patients

Until February 16, 2017, the median follow-up time was 32 (interquartile range [IQR]: 20.3–38.4) months (range, 0.7–47.1 years). During the tracked period, 20 patients died. The total annual mortality rate was 12.8%. Among patients who were dead, the median survival was 5.4 months (IQR: 2.2–17.8). In patients who died during the tracked period, the median survival was only 5.4 (IQR: 2.2–17.8) months; three of them died in the hospital, 17 (85%) died at home, Seventeen (85%) of them died at home, among whom the median interval from discharge to death was 8.4 months (IQR: 2.0–18.2) and the median time from discharge to death was 8.4 (IQR: 2.0–18.2) months. The cumulative survival curve (Fig. 2) showed that 14 patients died in the first year, accounting for 70% of all deaths, and 10 died in the first 6 months, accounting for 50% of all deaths.

Among the 47 survivors, 9 cases said they had been cured, 17 cases were continuing treatment with some “old and less active” anti TB drugs, the other 21 cases had given up treatment or just accepted palliative care such as nutritional support and cough relieving, only 12 of them said they visited the hospital regularly for examination and follow-up.

### Mortality associated factors

In univariable models, age > 50 years, history of smoking, BMI < 18.5 kg/m^2^, combined with the underlying disease, hemoglobin (Hb) concentration < 115 g/L, and albumin (ALB) < 35 g/L at admission were associated with increased risk of death. In multivariable models, lower BMI (adjusted hazard ration [aHR] *=* 4.5, 95% confidence interval [CI]: 1.3–15.7), history of smoking (aHR = 4.7; 95%CI: 1.7–13.2), and combined with the underlying disease (aHR = 3.5; 95%CI: 1.3–9.4) remained statistically significant (Table 1).

**Table 1:** Survival Analysis of 67 XDR-TB Patients

Variables	No. of cases, n (%)	Univariate Analysis		Multivariate Analysis	
		HR (95%CI)	*P*	aHR (95%CI)	*P*
Age>50 years	32(47.8)	2.82(1.08–7.35)	0.034	2.40(0.84–6.85)	0.103
Male sex	51(76.1)	1.32(0.44–3.96)	0.619	–	
BMI < 18.5 kg/m^2^	40(59.7)	4.30(1.26–14.72)	0.020	4.52(1.31–15.65)	0.017
Hb < 115 g/L	19(28.4)	2.49(1.03–6.03)	0.043	2.08(0.83–5.20)	0.117
ALB < 35 g/L	24(35.8)	4.37(1.73–11.03)	0.002	1.07(0.27–4.20)	0.920
TB course>3 years	32(47.8)	1.51(0.62–3.68)	0.369	–	
Retreatment TB cases	53(79.1)	0.43(0.17–1.08)	0.073		
Smoking history	27(40.3)	3.83(1.51–9.73)	0.005	4.67(1.66–13.16)	0.004
Drinking history	10(14.9)	2.18(0.79–6.01)	0.132		
combined with underlying diseases	23(34.3)	5.16(2.05–13.00)	0.001	3.48(1.30–9.36)	0.013

Definition of abbreviations: *XDR* extensive drug-resistant, *TB* tuberculosis, *HR* hazard ration, *CI* confidence interval, *aHR* adjusted hazard ration, *BMI* body mass index, *Hb* hemoglobin, *ALB* albumin

## Discussion

In our study, A total of 67 patients from four large-scale Tuberculosis Specialized Hospitals were first confirmed as XDR-TB cases and tracked for a median follow-up period of 32(IQR: 20.3–38.4) months. The data showed an overall mortality of 128 per 1000 person-year, and the median survival of deaths was only 5.4 (IQR: 2.2–17.8) months, which was significantly more serious than that reported in China [11, 12]. Smoking, BMI < 18.5 kg/m^2^ and the situation on combining of underlying diseases were significant predictors of mortality in this study. We also found 15% of deaths (3/20) were classified as died in the hospital, other 85% died at home, and the median interval from discharge to death was 8.4 (IQR: 2.0–18.2) months. In addition, 58(86.8%) patients remained positive for sputum smear or culture when discharged, indicating potential transition in the community, which was worthy of our further study and research.

To our knowledge, this is the first study in China to evaluate the mortality and survival analysis of inpatients with XDR-TB using active tracking. In this study, 85% of deaths were captured through telephone interview or home visits rather than medical record review, which supported our speculation that passive collection of vital status information would lead to considerable underestimation of mortality. In our study, 20 deaths occurred among 67 patients enrolled, translated into an overall mortality of 128 per 1000 person-year. The mortality greatly exceeds the relevant data in the previous literature and also exceeds the mortality of total HIV-negative patients with TB in China, which was reported 3.6(2.4–5) per 100,000 person-year in the 2017 Global Tuberculosis Report [1]. Obviously, survival situation of Chinese patients with XDR-TB is a crucial parameter to understand the related disease burden and calls for urgent actions to improve XDR-TB case management.

Among patients who were dead during tracking period, the median survival time was only 5.4 (IQR: 2.2–17.8) months, The findings suggested an alarming situation of XDR-TB patients in China. The cumulative survival curve showed that 14 patients died in the first year, accounting for 70% of all deaths, and 10 died in the first 6 months, accounting for 50% of all deaths. The data are similar to those reported in South Africa [17] and suggests that the first year (especially the first half year) after initial diagnosis of XDR-TB was responsible for the high risk of death. Patients in this period must be fully treated and given adequate attention to survive the high-risk period and win the time for the following treatment. Fan L et al. [5] found 49 deaths in 107 patients with XDR-TB in three provinces of South Africa after the follow-up of 24 months; the mortality rate (46%) was significantly higher than that in our study. Sheela [18] reported that the median survival (34 days [IQR: 18–90]) of XDR-TB patients who died within 180 days were also significantly shorter than that in our study. Excluding the lower living and medical conditions and other factors, we considered that the group of patients in our study ruled out the case of XDR-TB combined with HIV infection, which may greatly raise the mortality rate. It had been proven long ago that TB and HIV co-infection in South Africa was more than 70%, and XDR-TB combined with HIV patients had a long-term survival rate of less than 20% [6, 7].

Baseline clinical variables, including BMI < 18.5 kg/m^2^ and the combination of underlying diseases, were significant predictors of mortality in this study. Our findings are in line with those of a study from Korea in which XDR-TB patients with BMI < 18.5 kg/m^2^ had higher mortality [19]. Tang et al. [11] also demonstrated that BMI < 18.5 kg/m^2^ or comorbidities were an important factor in the poor prognosis of MDR-TB and XDR-TB. The implication of these findings is that patients with risk indicators should be given specific attention, and supportive intervention should be considered. We also found that XDR-TB patients with smoking history had higher mortality. The finding is potentially significant due to the high smoking rate in China and other countries. According to a territory-wide treatment program in Hong Kong, [20] smoking contributed to 16.7% of unsuccessful treatment outcomes after 2 years of follow-up and affected baseline disease severity, relapse, and bacteriologic response. As a result, smoking should be forbidden in DR-TB patients, especially in XDR-TB groups.

The study found that 63% of the XDR-TB patients were referred from off-site, 85% returned to the community, only 47 patients (70%) survived. Among the 47 survivors, only 12 patients said they visited the hospital regularly for examination and follow-up, we therefore infered that other patients did not accept DOT (Directly Observed Treatment, DOT) and standardized management at home. As expected, the study found that majority of the survivors only accepted some “old and less active” anti TB drugs or palliative care or even gave up treatment after being diagnosed as XDR-TB because of lack of effective therapeutic agents and methods, or economic difficulties, etc. The data from our tracking show that these 85% of patients survived in the community for 8.4 (IQR: 2.0–18.2) months, and in the course of 8.4 months, they lacked strong medical treatment conditions, awareness, and management conditions to control transmission in families and communities. So, the resistant strains can be transmitted repeatedly and widely in their daily activities, as well as in the process of the medical treatment and referral. These findings have brought great risks and challenges to our intervention and management, [21] and calls for rapid roll out of and access to the new drugs. The study also found that 21% of XDR-TB patients were new cases, who had denied having any previous anti-TB treatment and could be diagnosed as “transmitted resistance” only by reviewing the medical records. This result suggested that the spread of XDR-TB in China cannot be ignored. At present, the spread of MDR-TB and XDR-TB strains is a serious problem all around the world [22, 23]. Although the proportion of “transmitted resistance” in this study was significantly lower than that reported by South Africa (69.3%) [17] and Shanghai (54.5%) [24]. We anticipate that some patients who were diagnosed as “acquired resistance” by reviewing the history of anti-TB treatment in medical records might be un-detected DR-TB patients due to unavailability of DST upon the point when they were first diagnosed as TB and provided with anti-TB treatment for susceptible TB patients. We therefore reinforce the importance of universal access of DST for both new and retreatment TB cases, which is already stated in China National TB Program (NTP). Although there is no difference on treatment strategy for transmitted versus acquired XDR-TB, and no significant difference in mortality and survival between the two (HR = 0.43, 95%CI: 0.17–1.08, *P* = 0.073), interventions for preventing transmitted versus acquired disease differ. Acquired drug resistance can be decreased by ensuring completion of treatment and providing effective treatment. Stopping transmission requires determining and separating infectious patients, promptly initiating effective treatment, and improving ventilation in congregate settings. Therefore, the effective management of drug-resistant patients and regular treatment are equally important.

There are some limitations to this study. First, because patients were enrolled from four TB care centers, caution should be taken when the findings of this study are extrapolated into another setting in China. The resistance prevalence observed is likely higher than the nationwide average, representing a more serious situation. Second, the study included patients admitted between March 2013 and February 2015. Some patients were followed for only 2 years, and the time for observing the outcome was possibly short. Third, the study found that 21% of XDR-TB patients were new cases and the other 79% of the patients went to the referral hospital on a relatively advanced stage of the tuberculosis, which may lead to underestimation of survival time. In addition, Some studies, we compared mortality with which even in China or South Africa, were reported mortality during treatment cohort, while partial XDR-TB patients in our study were untreated, which could lead to overestimate the real mortality than the treatment cohort. But since XDR-TB lacks effective therapeutic regimens and drugs worldwide currently, we think the ratio of overvaluation will not be obvious.

## Conclusions

Our study suggested an alarming situation of XDR-TB patients in China with a high mortality rate, a sizable proportion of newly transmitted cases, and a long period in community. This observation calls for urgent actions in China to improve XDR-TB case management in China, including providing regimens with high chances of cure and palliative care, and enhanced infection control measures. In addition, XDR-TB patients with BMI < 18.5 kg/m^2^, combined with underlying diseases and smoking history, were significant predictors of mortality and should be given special attention.

## Abbreviations

aHR: Adjusted hazard ration
ALB: Albumin
BMI: Body mass index
CI: Confidence interval
DOT: Directly observed treatment
DR: Drug-resistant
DST: Drug susceptibility testing
Hb: Hemoglobin
HIV: Human Immunodeficiency Virus
HR: Hazard ration
IQR: Interquartile range
MDR: Multi-drug resistant
NTP: National TB Program
SD: Standard deviation
TB: Tuberculosis
XDR: Extensive drug-resistant
